# Supplementary material for: Predicting Modifiers of Genotype-Phenotype Correlations in Craniofacial Development
Source: Int J Mol Sci. 2023 Jan 8;24(2):1222. doi: 10.3390/ijms24021222 (PMC9864425; doi:10.3390/ijms24021222)
Supplement: Supplementary file 1 [file ijms-24-01222-s001.zip › ijms-2083010-supplementary.pdf]

Chemical	CAS Registry Number
Dihydroergocristine	24730-10-7
Piribedil	3605-01-4
Zalcitabine	7481-89-2
Pyrrolidine dithiocarbamate	25769-03-3
PCO-400	121055-10-5
Omeprazole	73590-58-6
Venlafaxine	93413-69-5
Trimipramine	739-71-9
Formestane	566-48-3
Salubrinal	405060-95-9

Supplementary Table S1. Randomly selected chemicals from the LINCS output that were tested.

Gene	Purpose	Forward (5' -> 3')	Reverse (5' -> 3')
gata3	RFLP	GGAAACAGAAGGGGATGGGG	TCTTACTAGAGAAGTGTAAGACAGCTAGGG
rpl8	qPCR housekpeeing	GGCTTATGCACCGTAAAG	TCCTCCGCCGGCCTCCCC
sfrp1a	qPCR	CCTACAAAAGATGATAAGCTCCGG	CAAGCAATATTGAACAACGCAAGG
gpc1a	qPCR	ATCGGCGATATAAACAGAGAAACG	GCTGATTACTCTTGTTGGTGTGGT
wnt7b	qPCR	TTATGGCATAGTCGTCCGCGGAG	CACTCTGGTAACGCTTATGGTCC
nkd1	qPCR	ATAGCACATTCATCGCATCCGGGTA	TGCGCTCTATTTGACGATCCTTTGG
nkd2a	qPCR	ATTAAAGCGACTGCACTACTGT	AAGGTCCGTCACGCAGACGACG
col2a1a	qPCR	TCAACCTGTACGGGAACTTTC	TATATCGTTCTCGGACGGAGA
cnmd	qPCR	GATAACTACAGTGCCGCTTACAGC	CCCTCTGTCGTCGCCGACGTCTGT
ccl25b	qPCR	AATATAGCCTTATTGTGATTCCAC	CCTATTGAGGCATTGACTGATGCG
col9a3	qPCR	GAAAGAGATCTGAAATGAACTGGT	CTATGTGACAGAAACTGTGCAAGT
ppp3r2	qPCR	ACCTAATCTCGTTAGTGTAGGTT	CTGACCGATACGTGCTTCGTTGA

Supplementary Table S2. Primers used for genotyping and qPCR experiments.

Chemical	Percentage with disrupted trabeculae
Predicted phenotype enhancers	
Flubendazole	4.65%
Rigosertib	6.41%
Etacrynic acid	8.33%
Clofibric acid	8.45%
Nocodazole	3.75%
Vinorelbine	7.84%
Vinblastine	7.69%
Vincristine	9.89%
Predicted phenotype suppressors	
PD-184352	2.56%
Triptolide	5.88%
Belinostat	0.43%
BMS-191011	3.71%
ISOX	6.73%
Daunorubicin	4.56%
Chromanol	6.98%
Alvocidib	5.78%

Supplementary Table S3. Percentage of wild-type embryos with disrupted (non-intact) trabeculae when exposed to predicted phenotype modifiers. The concentration used for each chemical is lowest observed adverse effect level (see Supplementary Figure S3).

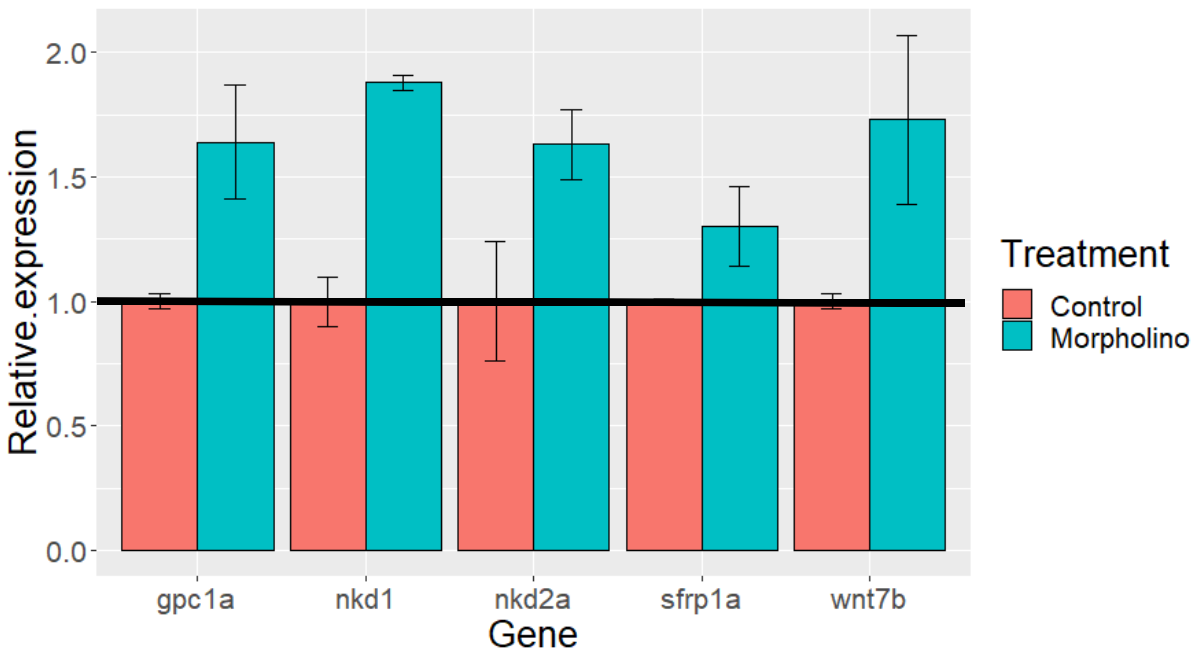

Relative.expression
2.0
1.5
1.0
0.5
0.0
gpc1a
nkd1
nkd2a
sfrp1a
wnt7b
Gene
Treatment
Control
Morpholino

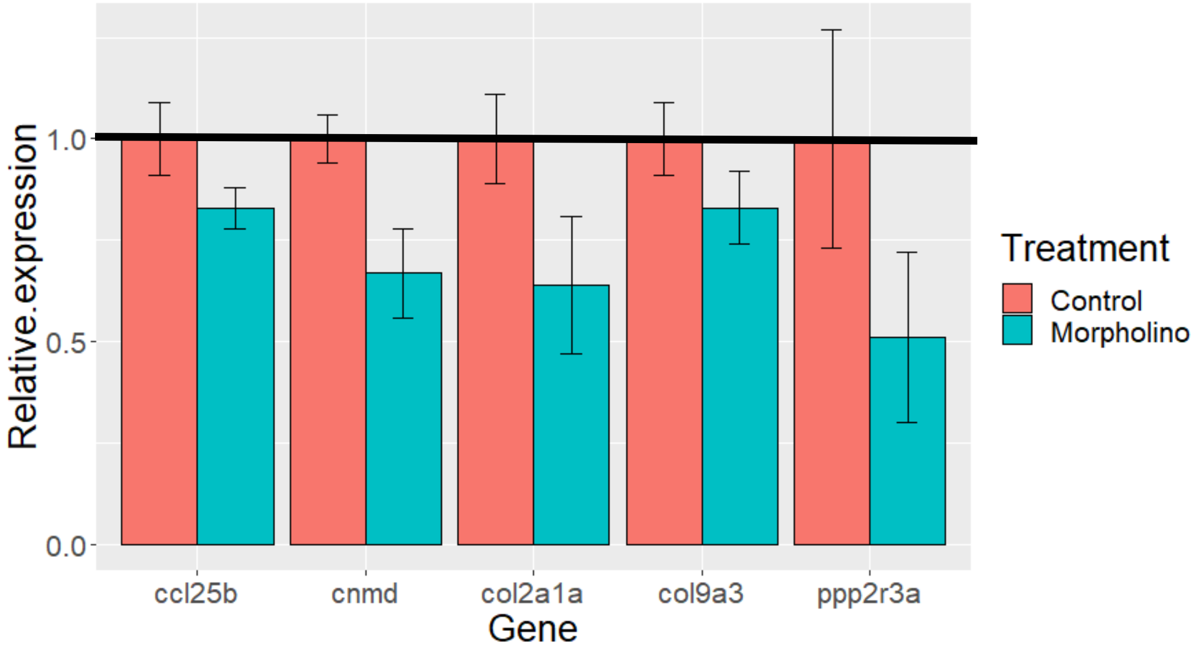

Supplementary Figure S1. qPCR validation of the five most up- and down-regulated genes from the RNA-seq dataset. Nine out of ten genes saw demonstrated significantly differential expression in the direction that was predicted from the RNA-seq data. Expression has been normalized relative to control samples.

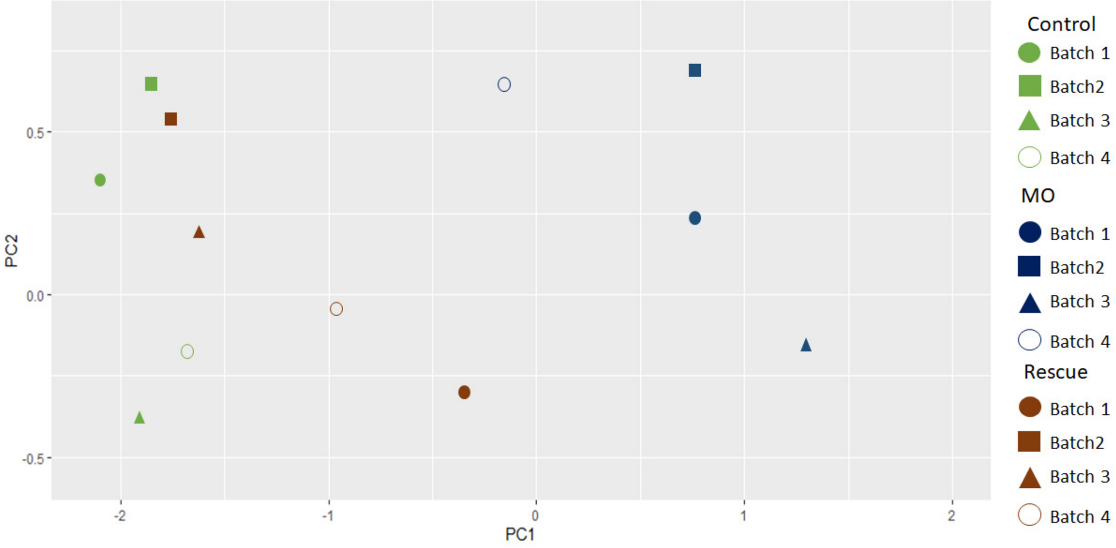

Supplemental Figure S2. Principal components analysis (PCA) of the RNA-seq samples. Principal component 1 (PC1) explains 35% of the transcriptional variance across datasets, and PC2 explains 17%. PC1 largely captured the difference between wild-type (and wild-type-like) phenotypes and mutant-like phenotypes.

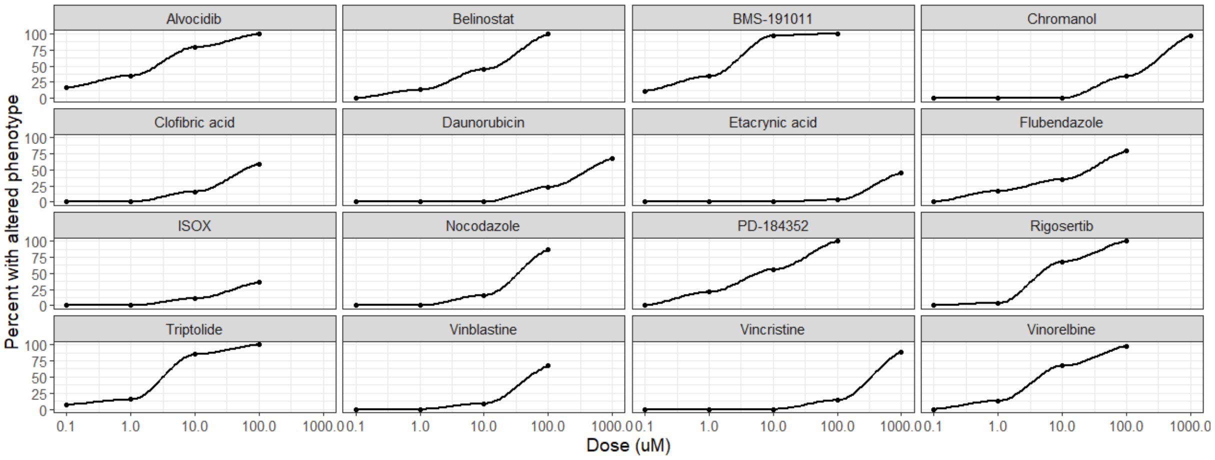

Supplemental Figure S3. A dose response curve (0.1 uM, 1 uM, 10 uM, 100 uM) in wild-type embryos was used to determine the lowest observed adverse effect level (LOAEL) that elicited a craniofacial defect.

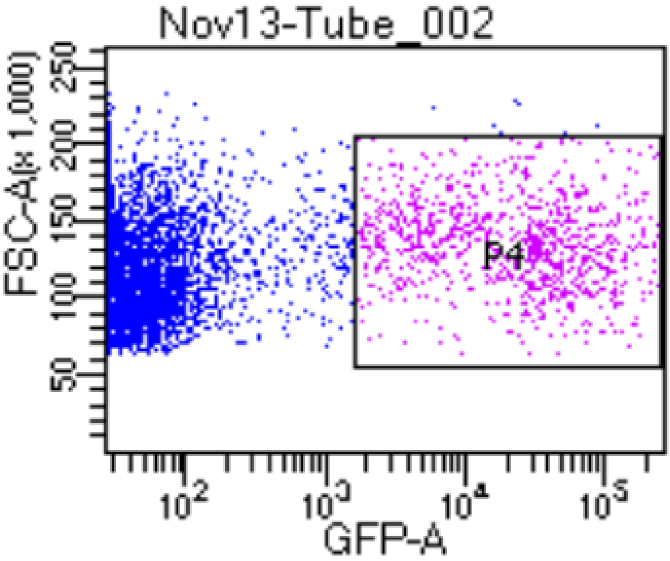

Supplemental Figure S4. Gating strategy to specifically collect fli1:EGFP positive cells. Separate sorts were done to determine the fluorescence of hsp70l:GATA3-EGFP and fli1:eGFP cells. The cells captured in the labeled box "P4" were specific to fli1:eGFP and this gate was used to separate that subset of GFP-positive cells in all subsequent sorts.
